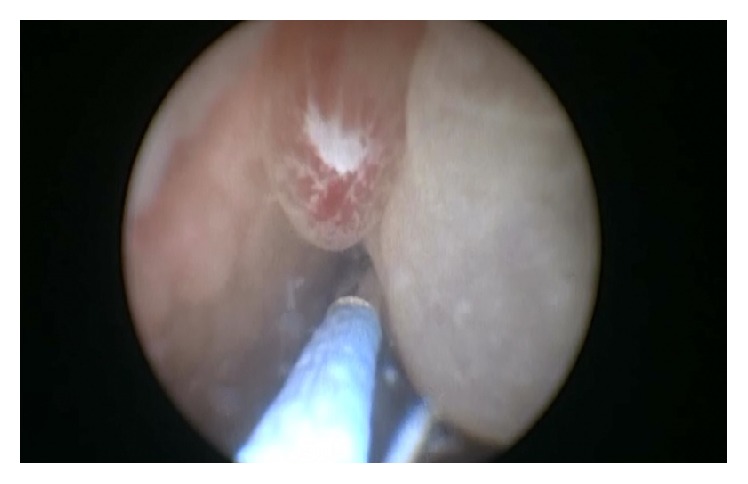# Mixed Botryoid and Spindle Cell Bladder Rhabdomyosarcoma: An Outstanding Pediatric Case

**DOI:** 10.1155/2017/8397826

**Published:** 2017-01-01

**Authors:** Tommaso Alterio, Roberto Chimenz, Salvatore Arena, Giovanni Conti, Sabrina Cardile, Carmelo Romeo, Carmelo Salpietro, Carmelo Fede

**Affiliations:** ^1^Department of Pediatric Sciences, University of Messina, Messina, Italy; ^2^Department of Pediatric Surgery, University of Messina, Messina, Italy

## Abstract

We report a case of a 3-year-old North African child, initially assessed for nonspecific urinary symptoms such as haematuria and burning urination. The ultrasound evaluation showed a vegetating mass occupying the lumen with weak vascular signs at the Colour-Doppler evaluation. An explorative cystoscopy was performed and it revealed a nonbleeding lesion, white in colour, pedunculated, projecting into the lumen, and associated with a brown satellite formation. Histological examination showed a mixed Botryoid and Spindle Cell Rhabdomyosarcoma. This mixed histology has not been described before and no statistical data are reported in literature so far. Despite the Embryonal Rhabdomyosarcoma variant being the most common, the association characterized by two histological Rhabdomyosarcoma subtypes such as Botryoid and Spindle Cell is rarely observed and it is important to get an accurate histological diagnosis in order to immediately start the correct treatment protocol.

## 1. Introduction

Rhabdomyosarcoma (RMS) is a malignant tumour derived from the embryonic mesenchymal cells that subsequently differentiate into striate muscle tissue [1]. According to the latest scientific literature, RMS represents 4–8% of the malignant tumours in pediatric age and most of them originated from the genitourinary tract, mainly in the bladder [2].

RMS includes a group of tumours characterized by three histological variants: embryonal RMS, alveolar RMS, and undifferentiated RMS. The embryonal RMS is divided into two different subhistological-types: Spindle Cell RMS and Botryoid RMS. Spindle Cell histology is typically found in paratesticular lesions whereas Botryoid subtypes are polypoid masses that fill the lumen of the bladder or vagina [3, 4]. Clinicians should be aware of the importance of the histological diagnosis in order to establish the most appropriate therapeutic regimen.

## 2. Case Presentation

**Figure 1:**
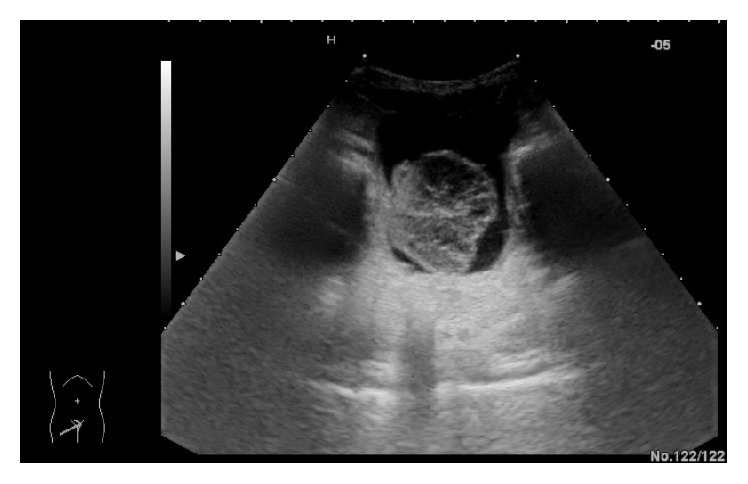


A 3-year-old North African boy was admitted to our Pediatric Nephrology Unit with a 4-day history of haematuria and burning urination. The physical examination was negative for pain, palpable mass in pelvic region, or other genitourinary (GU) symptoms. Blood tests showed white blood cell count (WBC) of 8,700/mm^3^ with lymphocytic predominance (68%); haemoglobin of 12.4 g/dl; platelet count of 339,000/mm^3^; C-reactive protein (CRP) and erythrocyte sedimentation rate (ESR) within normal limits. Urinalysis revealed a gold yellow colour; pH 5.5; specific gravity 1.020; proteins 30 mg/dl; plenty of red blood cells/High Power Field, pus cells 7–10/High Power Field. Urine culture was sterile. Ultrasound (US) scan of the bladder (Figure 1) documented a vegetating mass in the lumen with maximum size of about 40 × 41 mm, polylobed morphology and irregular contours, characterized by solid heterogeneous echogenicity and weak vascular signs at Colour-Doppler evaluation. Close to the above described mass, another sessile formation of about 6 mm was projected in the lumen. In light of that US aspect, an explorative cystoscopy was performed (Figure 2). It revealed a nonbleeding lesion, white-coloured, apparently pedunculated, projecting into the lumen next to the left anterolateral wall of the bladder, and associated with a satellite formation of brown colour. Because the cystoscopic features were not clear and an infective origin could not be excluded, the patient underwent an open biopsy. The histological report showed a pseudocystic, multilocular gelatinous, and moderately fluctuating formation of 52 × 45 × 11 mm and a brownish minute fragment of solid tissue of 7 × 7 mm. Both findings displayed features of mixed Botryoid and Spindle Cell type of Embryonal Rhabdomyosarcoma. The child was transferred to the pediatric oncology department to start on a chemotherapy cycle, according to RMS 2005 protocol of European Pediatric Soft Tissue Sarcoma Group (EPSSG) [5] in patients with standard risk, using IVA (ifosfamide, actinomycin D, and vincristine) associations.

## 3. Discussion

RMS is a malignant tumour of mesenchymal origin thought to arise from cells committed to a skeletal muscle lineage. Common sites of primary disease include the head and neck region, GU tract, and extremities [6]. Among the extracranial solid tumours of childhood, RMS is the third most common neoplasm after Neuroblastoma and Wilms' tumour [7]. Almost two-thirds of RMS cases are diagnosed in children <6 years of age although there is another midadolescence peak. It is slightly more common in males than in females (1.3–1.4 : 1) [7]. According to International Classification RMS is divided into three morphologic types: embryonal (with its Botryoid and Spindle Cell subtypes), alveolar, and undifferentiated [8]. Embryonal Rhabdomyosarcoma (ERMS) occurs in 55% of patients; the Botryoid variant occurs in 5% of patients; Alveolar Rhabdomyosarcoma (ARMS) occurs in 20% of patients and Undifferentiated Sarcoma (UDS) occurs in 20% of patients [5]. Mortality in RMS is highly related to age, site, and histology. The 5-year survival rate was highest in children aged 1–4 years (77%). Orbital and GU sites were the most favourable (86% and 80%, resp.). ERMS histology was best (67%) compared with ARMS histology (49%) [9]. The histological variant characterized by mixed component Botryoid and Spindle Cell, as in our case, has not been described before and no statistical data are reported in literature. Because it is very difficult to obtain a complete histological diagnosis of a mixed tumour through cystoscopic specimens and we did not know the behaviour of this particular subtype of RMS, we suggest always performing an open biopsy to ensure patients with mixed RMS are enrolled to a correct adjuvant therapeutic protocol. It is important to describe this peculiarity to understand whether this association may be associated with a different prognosis and whether it should be necessary to make changes to the basic treatment protocol used for Pediatric Rhabdomyosarcoma. Future reports will be able to clarify this aspect.

## Figures and Tables

**Figure 2:**